# A comparative analysis of using cage acrossing the vertebral ring apophysis in normal and osteoporotic models under endplate injury: a finite element analysis

**DOI:** 10.3389/fbioe.2023.1263751

**Published:** 2023-11-08

**Authors:** Jian Wang, Ziming Geng, Xiang Ma, Zepei Zhang, Jun Miao

**Affiliations:** Tianjin Hospital of Tianjin University, Tianjin, China

**Keywords:** biomechanical evaluation, surgical simulation, osteoporosis, lateral lumbar interbody fusion (LLIF), finite element analysis

## Abstract

**Background:** Lateral lumbar fusion is an advanced, minimally invasive treatment for degenerative lumbar diseases. It involves different cage designs, primarily varying in size. This study aims to investigate the biomechanics of the long cage spanning the ring apophysis in both normal and osteoporotic models, considering endplate damage, using finite element analysis.

**Methods:** Model 1 was an intact endplate with a long cage spanning the ring apophysis. Model 2 was an endplate decortication with a long cage spanning the ring apophysis. Model 3 was an intact endplate with a short cage. Model 4 was an endplate decortication with a short cage. On the basis of the four original models, further osteoporosis models were created, yielding a total of eight finite element models. The provided passage delineates a study that elucidates the utilization of finite element analysis as a methodology to simulate and analyze the biomechanical repercussions ensuing from the adoption of two distinct types of intervertebral fusion devices (cages) within the physiological framework of a human body.

**Results:** The investigation found no appreciable changes between Models 1 and 2 in the range of motion at the fixed and neighboring segments, the L3-4 IDP, screw-rod stress, endplate stress, or stress on the trabecular bone of the L5. Increases in these stresses were seen in models 3 and 4 in the ranges of 0.4%–676.1%, 252.9%–526.9%, 27.3%–516.6%, and 11.4%–109.3%, respectively. The osteoporotic models for scenarios 3 and 4 exhibit a similar trend to their respective normal bone density models, but these osteoporotic models consistently have higher numerical values. In particular, except for L3-4 IDP, the maximum values of these parameters in osteoporotic Models 3 and 4 were much higher than those in normal bone quality Models 1 and 2, rising by 385.3%, 116%, 435.1%, 758.3%, and 786.1%, respectively.

**Conclusion:** Regardless of endplate injury or osteoporosis, it is advised to utilize a long cage that is 5 mm longer on each side than the bilateral pedicles because it has good biomechanical features and may lower the likelihood of problems after surgery. Additionally, using Long cages in individuals with osteoporosis may help avoid adjacent segment disease.

## Introduction

The area of minimally invasive spine surgery has advanced significantly since Obenchain first laparoscopic lumbar discectomy in 1991 (Obenchain, 1991). The various advantages of minimally invasive treatments, including lessened postoperative discomfort, shortened hospital stays, and quicker return to normal activities, have attracted both surgeons and patients (Ozgur et al., 2006). Due to its low risk of complications, lateral lumbar interbody fusion (LLIF) through a lateral technique has gained popularity and been routinely used to accomplish interbody fusion (Elowitz, 2015; Walker et al., 2019).

However, these LLIF operations also carry the risk of different perioperative problems, such as internal fixation failure, nearby spinal degeneration, and vertebral endplate damage, much like other forms of lumbar fusion surgery (Zeng et al., 2018; Walker et al., 2019). Cage subsidence can potentially jeopardize stability and reduce fusion rates despite posterior screen-rod attachment. Numerous variables, such as bone quality or osteoporosis, endplate invasion during discectomy, high levels of bone morphogenetic protein, the presence or absence of additional fixation, cage design, and annular tension (preload) brought on by cage height, all affect the degree of sinking (Polly et al., 2000; Grant et al., 2001; Oxland et al., 2003). Additionally, research has shown that the area where the cage meets the spinal surface is another factor affecting cage subsidence (Yuan et al., 2020).

Endplate injuries commonly occur during the process of endplate preparation and cage implantation (Kim et al., 2021) and the incidence of these injuries typically ranges from 10% to 22% (Marchi et al., 2013; Malham et al., 2015). Such injuries may result in segmental lordosis and a reduction in the height of the intervertebral foramen, as well as the cage subsiding to the level of the neighboring vertebral endplate. Additionally, they could affect the postoperative indirect decompression effect, leading to an unfavorable outcome. Additionally, the frequency of osteoporosis among the senior population has significantly increased in recent years. As a result, osteoporosis is becoming more common among individuals who need lumbar interbody fusion surgeries (Song et al., 2022). Previous studies have consistently demonstrated that osteoporosis has a major impact on the lumbar spine’s biomechanics (Kang et al., 2022). This alteration in biomechanics increases the risk of vertebral fractures, failure of internal fixation, and subsidence of implants such as stents (Wang et al., 2021).

Contrary to the center of the endplate, which is supported by cancellous bone, the vertebral ring apophysis, which is composed of the surrounding cortical bone border, has been demonstrated to be the strongest area on the superior surface of the vertebral body (Grant et al., 2001; Grant et al., 2002). We have created two models for this finite element analysis: one uses a long cage that crosses the lumbar vertebral ring apophysis, and the other uses a short cage that only extends over the endplate. These models were applied to osteoporotic bone models as well as normal bone models with both intact and injured endplates. We sought to assess the biomechanical characteristics of these eight sets of models by measuring and examining the movements of bending forward and backward, bending left and right, rotating left and right. According to our theory, the long cage model would perform better biomechanically independent of the endplate’s integrity or the existence of osteoporosis.

## Materials and methods

**FIGURE 1:**
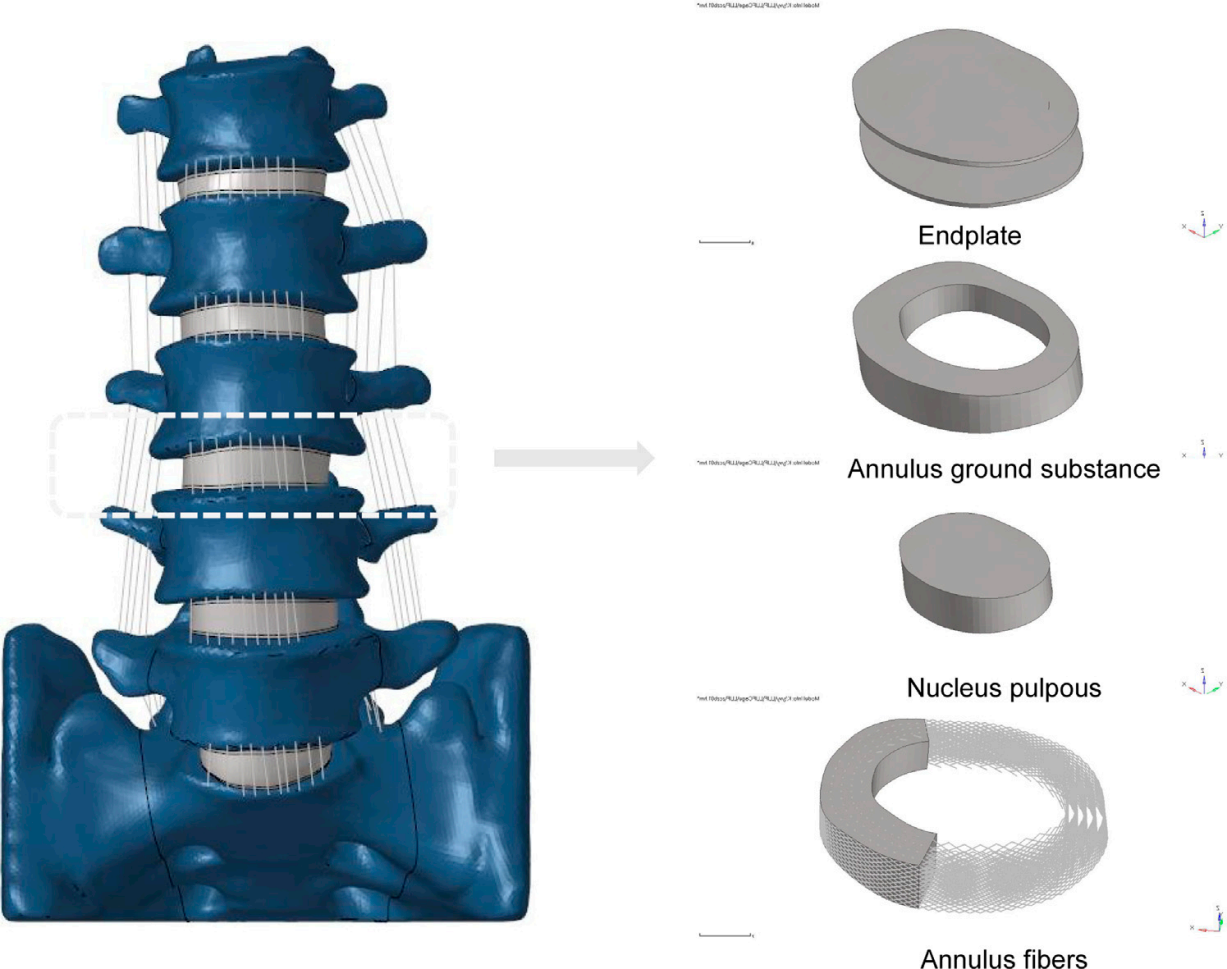
Established L1-S finite element model and its details (endplate, nucleus pulposus, annulus ground substance, annulus fibers).

The L1-S lumbar spine model was developed using data from a healthy adult male volunteer, as shown in Figure 1. The 28-year-old volunteer did not have a history of spinal diseases or injuries, according to clinical imaging testing. He was 173 cm tall and weighed 72 kg. The task of recruiting volunteers fell to the Department of Spine Surgery at Tianjin Hospital, and informed permission was acquired legally. The study protocol was sent to the Tianjin Hospital Ethics Committee for approval. The principles of the Declaration of Helsinki were strictly adhered to at all stages of the study procedure. We employed thin-slice CT imaging with a thickness of 0.625 mm to scan patients, capturing comprehensive images of their lumbar vertebrae and sacrum. The model reconstruction process followed the same procedures as an earlier experimental study (Wu et al., 2022). Utilizing mimics20 (Materials, Leuven, Belgium), the lumbar spine’s 3D geometric surface model was created and saved in STL format (Kim et al., 2014). The 3-Matic 12.0 software application from Materialise Inc. was used to process a 3D geometric model. This program included a number of features, such as wrapping, smoothing, and Boolean operations. To remove unnecessary triangular surfaces, the model underwent refinement, which produced three-dimensional pictures that were more elaborate and detailed. To ensure the precision of the simulation, the intervertebral discs and annulus fibrosus located within the anterior column of the spine, as well as the facet joints within the posterior column, were reconstructed (Alizadeh et al., 2013). The lumbar spine’s three-dimensional surface models were processed using Geomagic Studio 12.0, a program created by the North Carolina-based company Geomagic. In the processing, smoothing techniques were used to enhance the surface characteristics and guarantee the models’ correctness. The models that had been processed were then loaded into Altair’s Hypermesh2017 program, which is situated in Troy, Michigan, in the United States. Mesh structures for the models were created using meshing techniques in Hypermesh 2017. The corresponding seven ligaments of the spine were also reconstructed. The combined models and each of their associated characteristics were produced using Simulia’s Abaqus 2020 software, situated in Johnston, Rhode Island, USA, in order to finish the study. The Abaqus environment’s ability to do finite element analysis allowed for a thorough assessment of the lumbar spine models’ biomechanical characteristics and behavior (Li et al., 2014; Su et al., 2018).

A 3D finite element model in its normal form was painstakingly recreated for this scientific project. An intricate hexahedral mesh structure was used to accurately represent the intervertebral disc, which is an essential part of the model. The nucleus pulposus, the fibers of the annulus fibrosus, the endplate, and the annulus fibrosus matrix were all perfectly depicted by this mesh structure. Both the top and lower endplates were given a constant thickness of 0.5 mm to correctly represent the properties of the endplates. It is also important to remember that the nucleus pulposus made up a sizeable section of the disc, namely between 30% and 40% of the overall disc area. To guarantee the finite element model’s integrity and correctness in portraying the typical lumbar spine structure, these factors were taken into account when it was being built (Polikeit et al., 2003; Choi et al., 2017; Zhao et al., 2018; Lu and Lu, 2019). Cortical bone and articular cartilage have thicknesses of 1 and 0.2 mm, respectively, and the truss element is an ideal option for reproducing these parts because it was made specifically to withstand tensile stress (Kim et al., 2014; Choi et al., 2017). The annulus fibrosus, a crucial part of the intervertebral disc, was meticulously constructed with five layers, organized from the innermost to the outermost. At a tilt angle of around 30°, each layer was accurately inserted into the annulus fibrosus matrix. The elastic strength increased proportionally from 360 MPa in the inner layer to 550 MPa in the outermost one (Schmidt et al., 2007; Lu and Lu, 2019). The whole L1-S model, developed using material attributes in accordance with previously documented literature, has 1011182 units and 248371 nodes (Table 1) (Huang et al., 2016; Choi et al., 2017; Lu and Lu, 2019; Su et al., 2020). The vertebral body adopts a tetrahedral structure, and the endplate, nucleus pulposus and matrix adopt a hexahedral structure.

**TABLE 1:** Material properties used by finite element model.

Component	Young’s modulus (MPa)	Poisson ratio	Cross-sectional Area (mm2)
Vertebra			
Cortical bone	12,000	0.3	
Cancellous bone	100	0.2	
Posterior element	3,500	0.25	
Sacrum	5,000	0.2	
Facet	11	0.2	
Disc			
Endplate	24	0.4	
Nucleus pulpous	1	0.49	
Annulus ground substance	2	0.45	
Annulus fibers	360–550		0.15
Ligaments			
ALL	7.8		63.7
PLL	10		20
LF	15		40
CL	7.5		30
ISL	10		40
SSL	8		30
ITL	10		1.8
Implants			
Cage (titanium alloy)	110,000	0.3	
Bone graft	100	0.2	
Screws and rods (titanium alloy)	110,000	0.3	

### Model simulation

**FIGURE 2:**
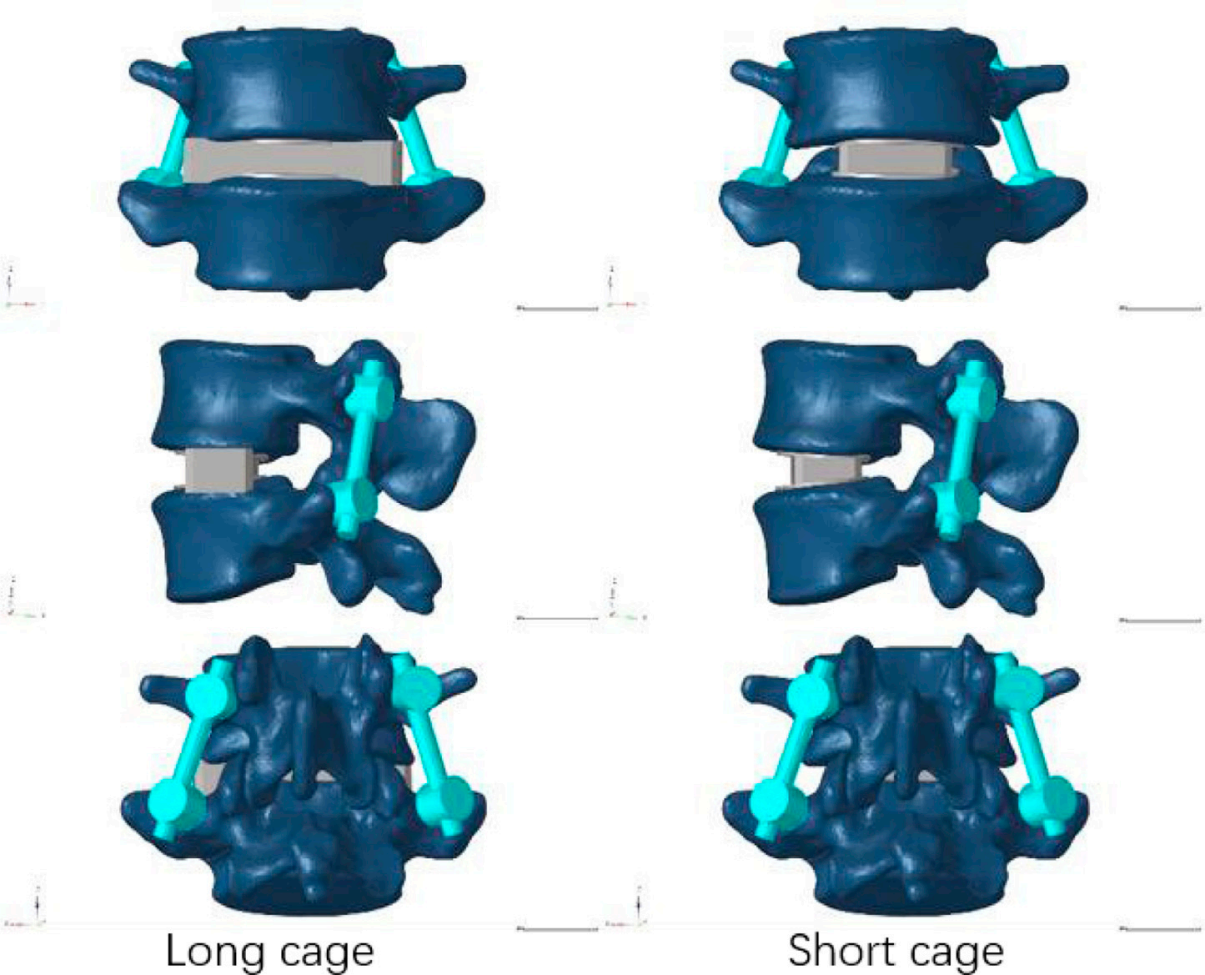
Models after lateral fusion of the two cages.

Our research concentrated on performing lateral lumbar interbody fusion (LLIF) primarily in the L4/5 segment because to the prevalence of lumbar degenerative disease in this region (Buser et al., 2021). Following the well-known Weinstein’s protocol, we painstakingly inserted four pedicle screws in the L4 and L5 vertebrae to support the injured region (Weinstein et al., 1988), and then the intervertebral disc at the L4-5 level was removed. During this test, We utilized Pro/Engineer software to fabricate two distinct types of cages. Nearly identical in size as the endplate, the short cage was made of titanium alloy and covered the space directly above it (Briski et al., 2017). The dimensions of the long cage in this study, 56 mm*18 mm*12 mm, and the short cage, 30 mm*18 mm*12 mm, were based on the actual model situation (Alimi et al., 2018; Kotheeranurak et al., 2021). Figure 2 depicts the model of the screen-rod system, which included two connecting rods with a diameter of 5.5 mm, four pedicle screws with a width of 6.5 mm, and a length of 45 mm.

**FIGURE 3:**
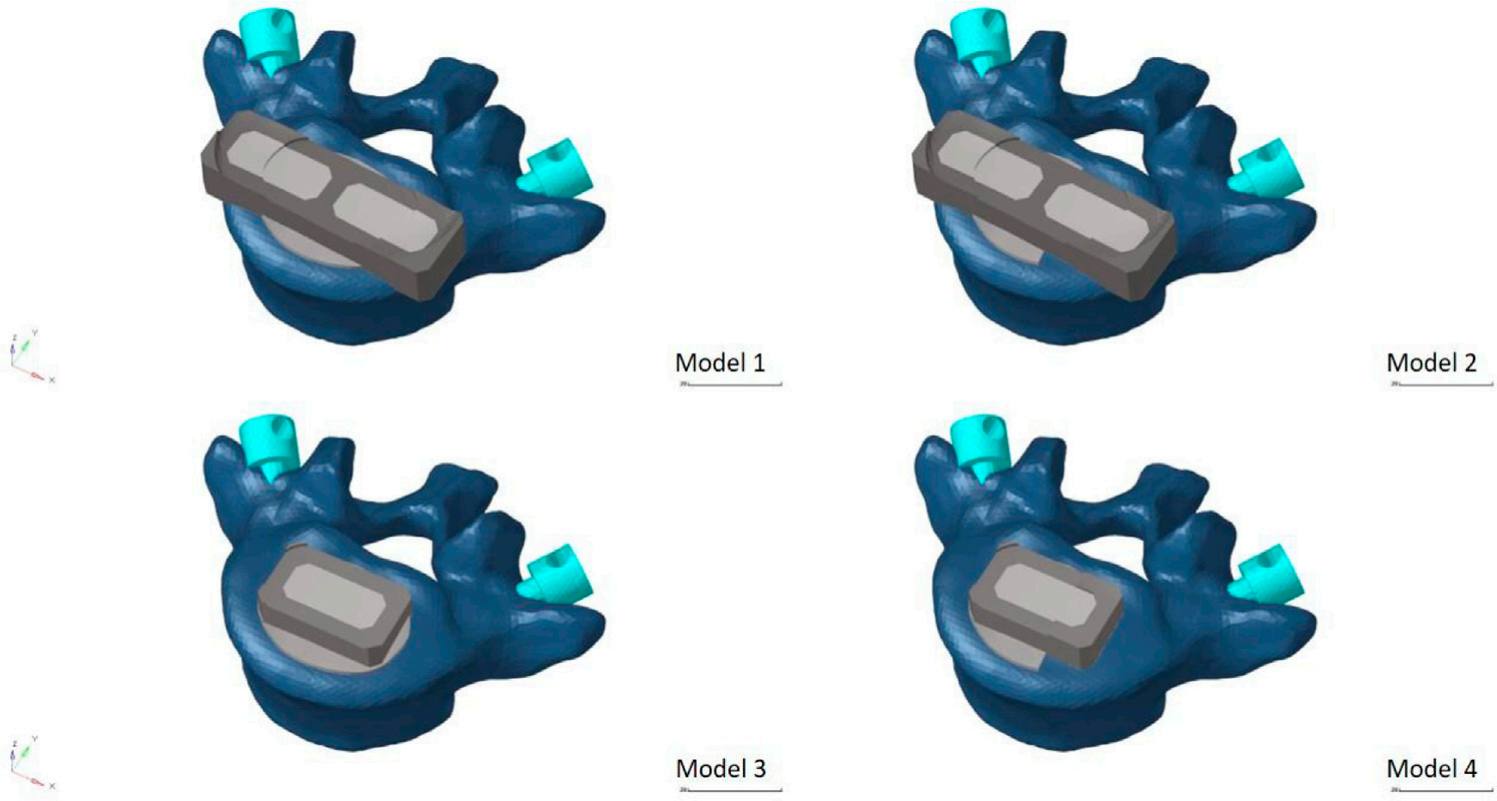
Model with two cages after simulated endplate failure. Model 1: intact endplate with long cage spanning the ring apophysis; Model 2: endplate decortication with long cage spanning the ring apophysis; Model 3: Intact endplate with short cage; Model 4: endplate decortication with short cage.

In order to mimic the circumstances of endplate damage, we treated the upper endplates of the L5 vertebrae in eight models, as shown in Figure 3. We employed binding commands to limit movement between the cage and the vertebral body to simulate stable fusion after surgery, as did the screen-rod system (Liang et al., 2020; Sengul et al., 2021). It is crucial to note that this experiment did not account for screw slippage within the vertebral body. The screw threads were cut out of the analysis to speed things up without affecting the study’s conclusions (Liu et al., 2020; Han et al., 2021). To replicate osteoporotic features, we altered the values of several model components while keeping other variables constant. This was done for the osteoporotic model (Park et al., 2013; Cho et al., 2015) (as shown in Table 2).

**TABLE 2:** Comparison of material properties between normal and osteoporotic models.

Component	Young’s modulus (MPa)	Poisson ratio
Normal Model		
Cortical bone	12,000	0.3
Cancellous bone	100	0.2
Posterior element	3,500	0.25
Endplate	24	0.4
Osteoporotic Model		
Cortical bone	8,040	0.3
Cancellous bone	34	0.2
Posterior element	2,345	0.25
Endplate	16.08	0.4

### FE model validation

We used the validation method of Renner et al. (Renner et al., 2007): First, the range of motion of the whole sacral base was entirely reduced to zero in all directions. To replicate the motion of the lumbar spine in normal life, six pure bending moments—eight Nm in flexion, six Nm in extension, six Nm in left and right lateral bending, and four Nm in left and right rotation—were applied to the center of the top surface of L1. We confirmed the disc pressure (IDP) at the L4/5 level in addition to the range of ROM at each lumbar level: We evaluated the IDP of the L4/5 segment by gradually applying compressive loads (300N, 1000N), building on previous study by Brinckmann et al. (Brinckmann and Grootenboer, 1991).

### Boundary and loading conditions

**FIGURE 4:**
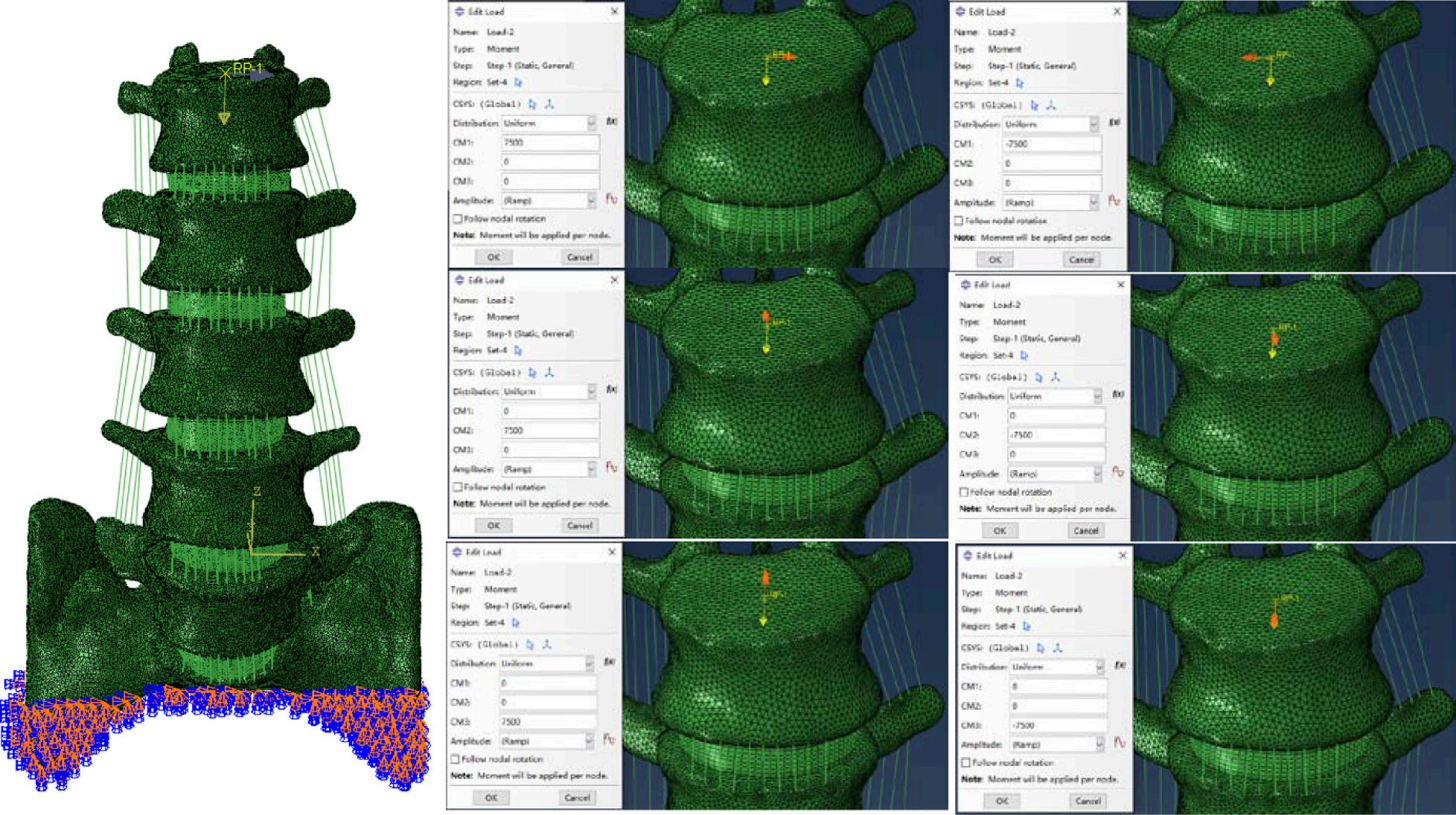
Schematic representation of the boundary conditions of the model.

The ABAQUS application was used for the analysis and computational assessment of the completed model. In order to put the whole thing together, the INP format of each model component was first imported. The appropriate boundary conditions were then established, and loads were applied concurrently. To simulate the physiological weight borne by the lumbar spine, a 280Nm moment was applied vertically downward to the geometric center of the L1 upper surface (Choi et al., 2017; Takenaka et al., 2020). Then a bending moment of 7.5 N m was applied simultaneously at the points set above to simulate the motion in six directions, as shown in Figure 4 (Wu et al., 2022).

### Assessment indexes

The model’s data, including the ROM of the fixed segment and adjacent segments in six directions, the intervertebral disc pressure (IDP) of adjacent segments, the stress on the screw rod system, the stress on the natural and injured endplates, and the stress on the cancellous bone on the upper surface of L5, were all calculated using the Abqus software.

## Results

### FE model validation

**FIGURE 5:**
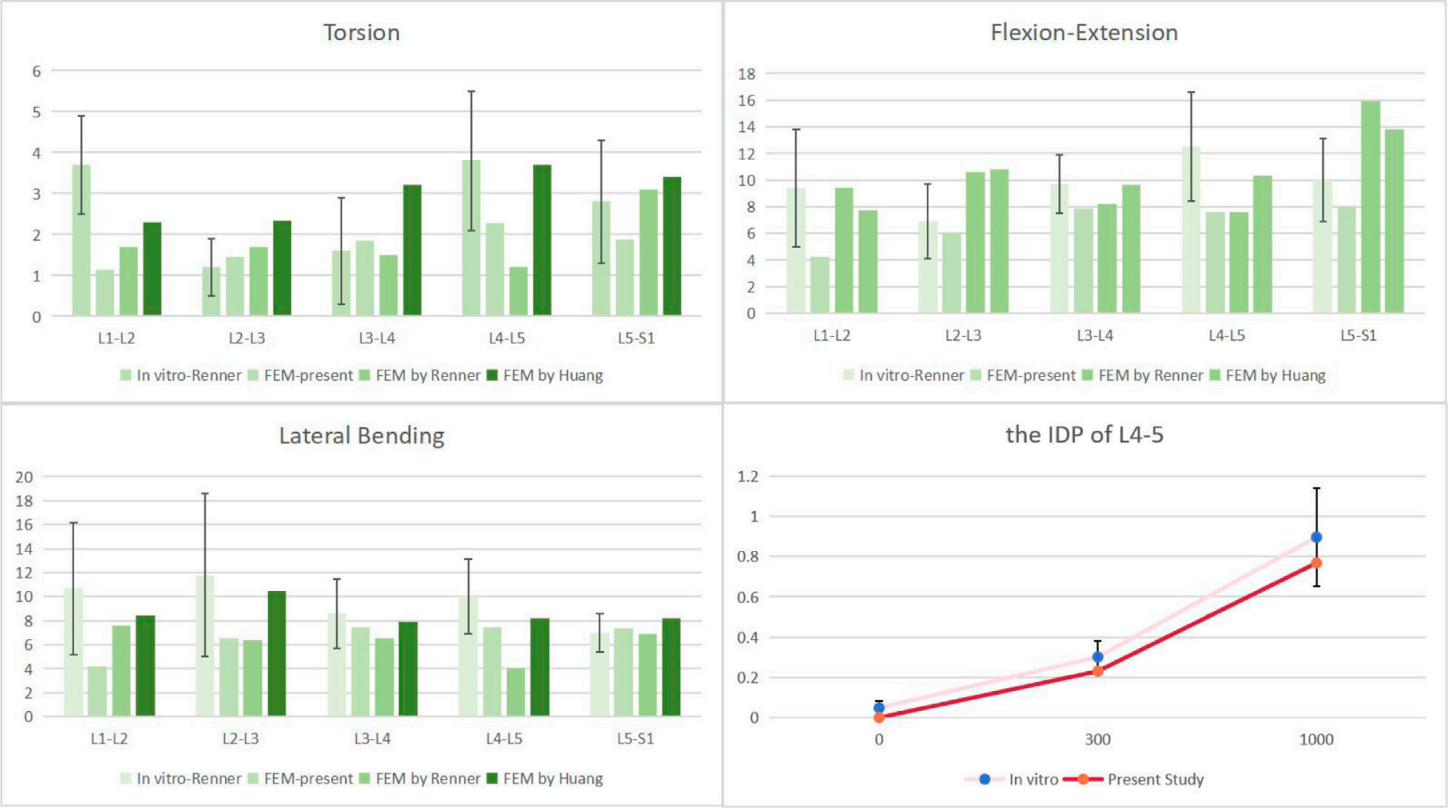
Comparison of the ROM of each motion segment and the IDP of L4/5 between the present and previous studies.

Firstly, the ROM of each segment was compared with previous finite element simulations and cadaver experiments (Brinckmann and Grootenboer, 1991; Renner et al., 2007; Huang et al., 2016), as shown in Figure 4. With the exception of the L1-2 segment, which was excluded from our experimental design, our results showed that the range of motion (ROM) for each segment was similar with results from other investigations. The remaining segments’ ROM values were within one standard deviation of the studies mentioned. At the same time, the verification of L4-5 segment IDP also conforms to one standard deviation only, as shown in Figure 5. As a result, we believe the finite element model utilized in this work will hold up to further examination.

### The ROM of the fixed segment

**FIGURE 6:**
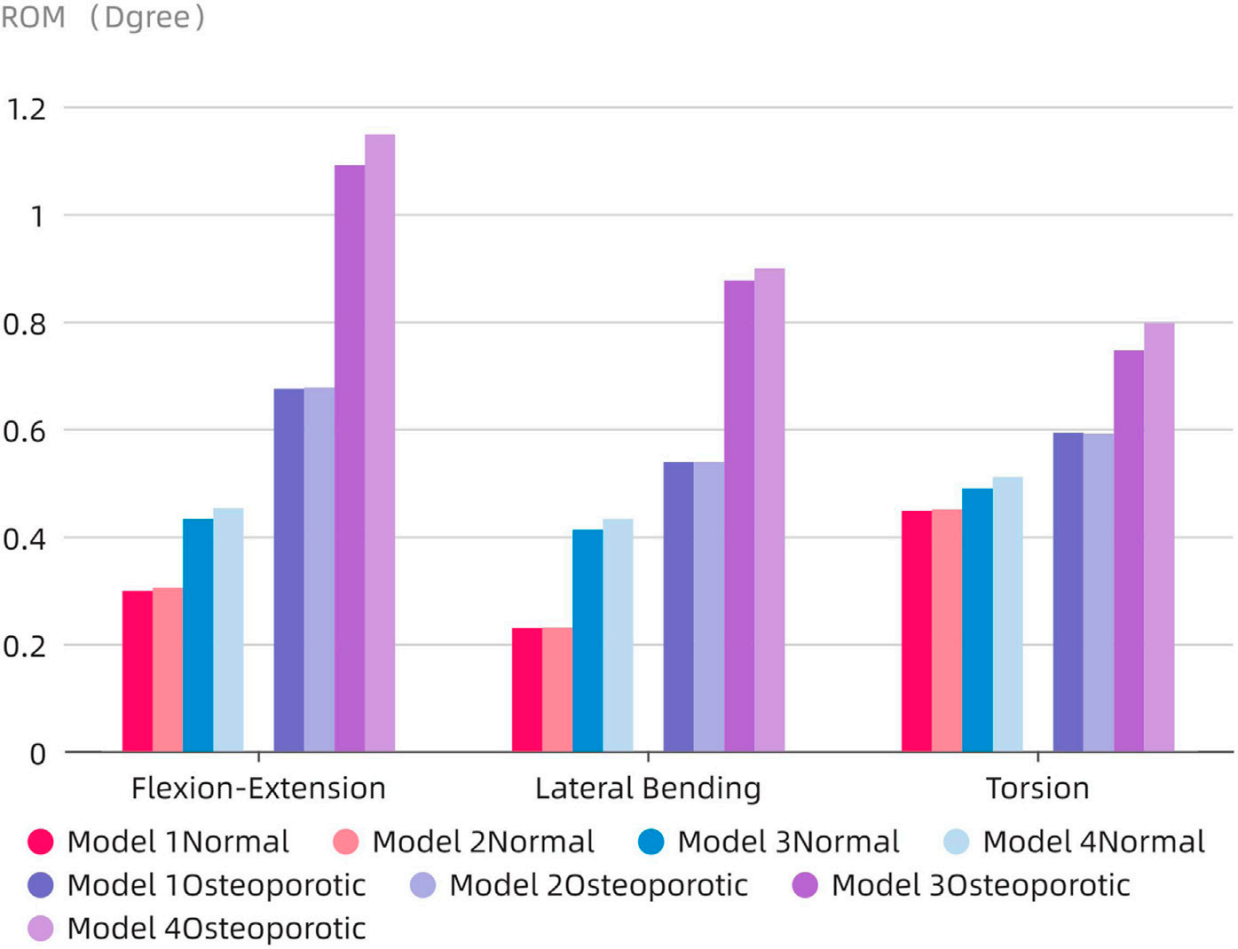
Comparison of the ROM at the fusion segment.

**FIGURE 7:**
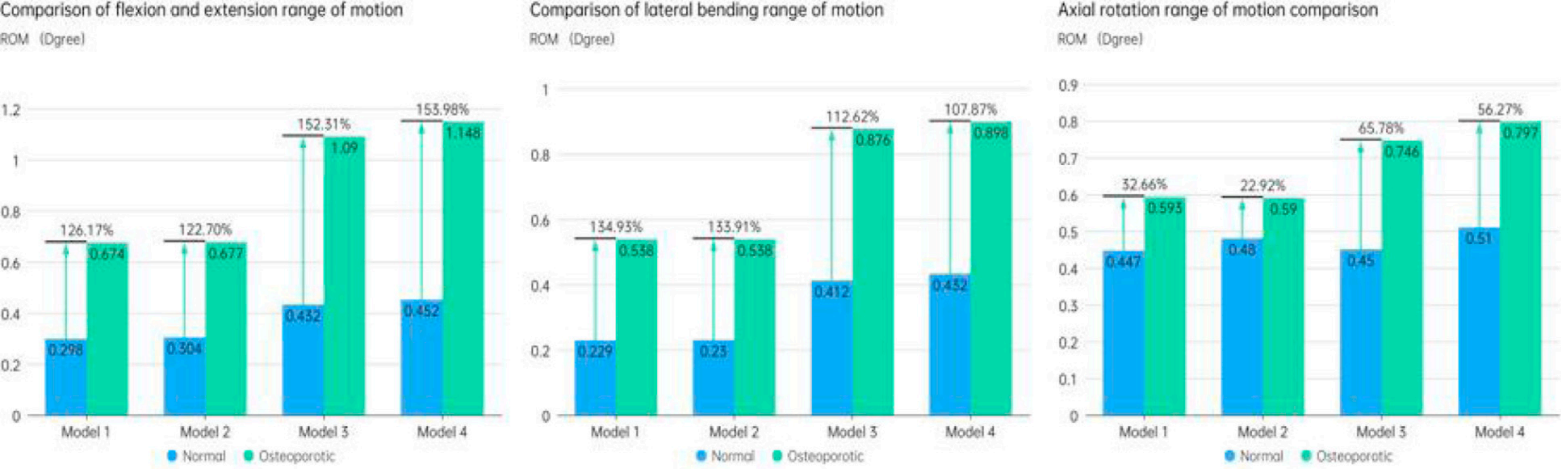
Comparison of ROM in the fixed phase between the normal model and the osteoporotic model.

The ROM of the fixed segment shown in eight surgical models and the intact model within the fused section is shown in Figure 6. The findings unmistakably show that the internal fixation device had a considerable stabilizing effect, resulting in a significant decrease in ROM in all six directions as compared to the whole model. The ROM values for flexion and extension for the Normal models (Models 1–4) were 0.298°, 0.304°, 0.432°, and 0.452°, respectively. 0.229, 0.23, 0.412, and 0.432° were the lateral bending ROM values, respectively. Similar results were found for the axial rotation ROM values, which were 0.447°, 0.48°, 0.45°, and 0.51°, respectively. The ROM values for flexion and extension in the osteoporotic models (Models 1–4) were 0.674°, 0.677°, 1.09°, and 1.148°, respectively. The axial rotation ROM values were 0.593°, 0.59°, 0.746°, and 0.797°, respectively, whereas the lateral bending ROM values were 0.538°, 0.538°, 0.876°, and 0.898°. Figure 7 compares the osteoporosis model to the normal model, emphasizing the contrasts. Notably, Model 4 (153.98% increase), Model 1 (134.93% increase), and Model 3 (65.78% increase) showed the greatest differences in all six directions between the osteoporosis and normal models, respectively.

### Range of motion and intra-disc pressure of adjacent segments (L3-4)

**FIGURE 8:**
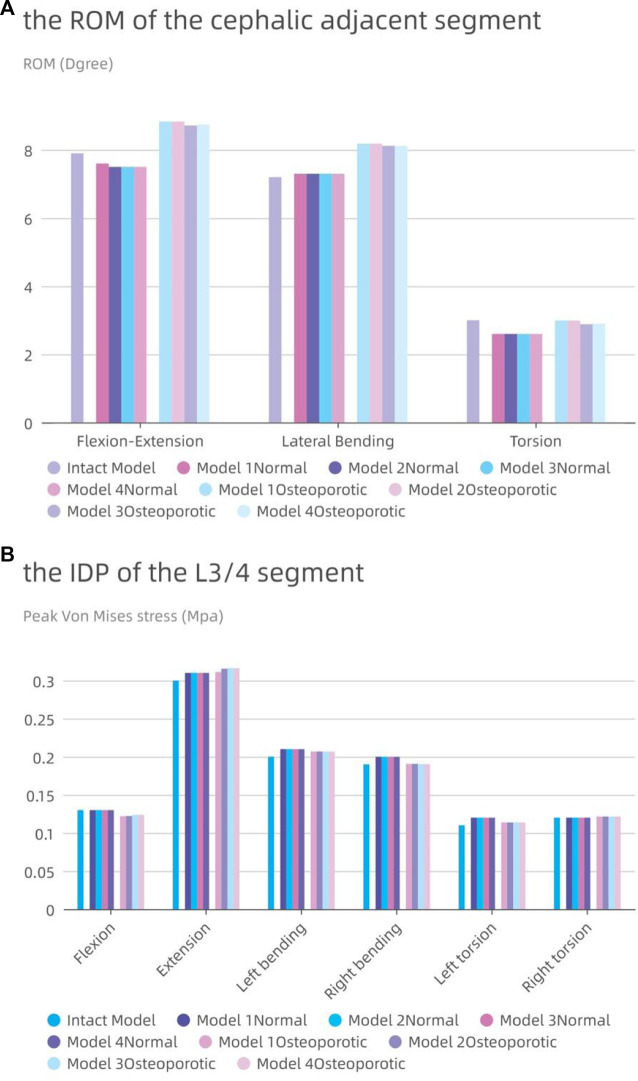
The intervertebral disc pressure (IDP) and range of motion (ROM) at L3-L4. **(A)** the ROM of L3-L4; **(B)** the IDP of L3-L4.

The accompanying Figure 8A displays the ROM of the L3-4 sgement for each model. Except for lateral bending, the ROM of the neighboring segments in the postoperative model of the normal model was often less than that in the intact model. The ROM of the postoperative model barely rose by 0.1° in comparison to the intact model, even when lateral bending was included. Contrarily, in the osteoporotic model, the range of motion (ROM) of the neighboring segments was larger than in both the intact and normal models, with flexion and extension motions showing the most obvious differences (an increase of 0.9° and 1.3°, respectively, compared to the intact and normal models).

In Figure 8B, the IDP of each model in the L3-4 segment is also shown. Although the difference was frequently not substantial, the IDP of the nearby segments was typically higher than or comparable to that of the entire model. With the exception of a modest rise of 0.01 MPa during posterior movement, the IDP of the L3/4 segment for the osteoporotic model was comparable to that of the normal model. The IDP of the L3/4 segments of the four osteoporotic models was comparable to that of the normal model, except for a slight increase of 0.01 MPa during posterior exercise. All results showed roughly the same trend in all eight models, with maximum IDP at L3-4 during posterior movement and minimum pressure during axial movement.

### Stress analysis of the internal fixation system

**FIGURE 9:**
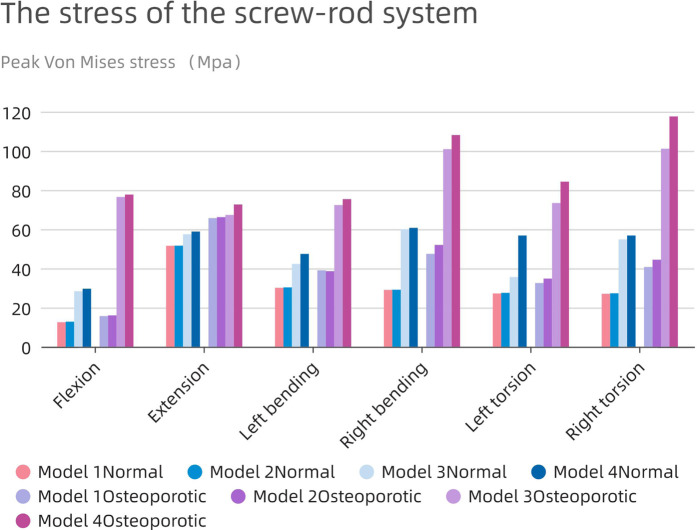
The stress of the screw-rod system.

Severe postoperative complications, such as screen-rod breakage and loosening, depend largely on the pressure of the screen-rod system. As shown in Figure 9, the change patterns of the two models are similar, and the stress on the nail-rod system of Model1 and 2 is less than that of Model 3 and 4. When the endplate is damaged, the overall screw rod stress will rise further.

The average maximum stress in the corresponding osteoporosis model is higher (63.34 MPa) than the average stress in the normal model (39.36 MPa), which is remarkable. The highest stress in the screw-rod system is shown in the normal model during rearward movement in the long cage operation model (51.6 MPa), and during right bending movement in the short cage operation model (60.7 MPa). In the whole experimental action, the maximum force of the screw rod system can be stimulated in the right rotation, up to 117.6 Mpa.

The use of the bigger cage results in decreased total stress in all directions as compared to the use of the smaller cage. Additionally, because the fusion device is larger than the endplate, the tension in the model’s screw-rod system is unaffected by the endplate’s impairment. The screw-rod system is more stressed when the intervertebral cage’s length diminishes, a tendency that is especially obvious in the osteoporotic model.

### Endplate stress of the fixed segment (L5 upper endplate)

**FIGURE 10:**
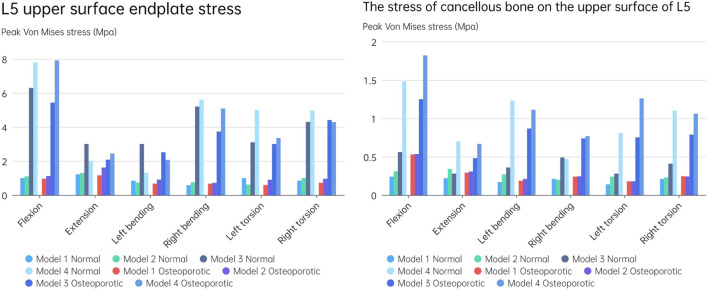
**(A)** L5 upper surface endplate stress; **(B)** The stress of cancellous bone on the upper surface of L5.

Figure 10A displays the endplate stresses for the eight models under investigation. The L5 upper endplate underwent substantially less stress in Models 1 and 2 with the enlarged cage than it did in the standard form. The backward movement of Model 2 produced the most stress, measuring 1.3 MPa, while the right-leaning movement produced the lowest stress, 0.58 MPa. In addition, during left rotation and left bending, the endplate stress of Model 2 was lower than that of Model 1. In contrast, overall endplate stress for the models with the short cage was much greater than for Models 1 and 2. The osteoporotic model’s endplate stress trend closely matched that of the healthy model. However, the osteoporotic model’s overall average maximum stress (2.39 MPa) was lower than the model with normal bone mass’s (2.6 MPa). Often speaking, the L5 upper endplate stress is successfully decreased by the long cage design, whereas the L5 upper endplate stress is often increased by the short cage design. It is significant to note that regardless of the existence of osteoporosis, endplate failure circumstances will usually result in increased stress on the surviving endplate.

### Cancellous bone stress on the upper surface of L5

Figure 10B depicts an evaluation of the stress on cancellous bone above L5 of using several models. The maximum stress for Models 1 and 2, which have typical bone quality, was 0.34 MPa. The Model 1 left rotation produced the lowest stress, which was measured at just 0.14 MPa. Between Models 1 and 2, the highest stress difference was 0.12 MPa. On the other hand, there was a noticeable tendency toward rising stress on cancellous bone in the models with the short cage, reaching up to 1.48 MPa. This pattern was particularly obvious when endplate damage was present. Notably, compared to Models 1 and 2, Model 3 showed an increase in stress of 0.32 and 0.27 MPa, respectively. Similar to Models 1 and 2, Model 4 saw a rise in stress of 1.24 and 1.17 MPa, respectively.

The stress pattern in the osteoporosis model matched that in the model with typical bone quality. When there occurred endplate failure, the greatest stress was recorded during flexion movement and reached 1.82 MPa. In comparison to the normal bone quality model, which has a maximum average stress of 0.45 MPa, the osteoporotic model of trabecular bone has a maximum average stress of 0.62 MPa. In comparison to models with short cages, those with lengthy cage designs often showed less stress. Overall, Models 1 and 2 showed much less stress on the top surface of L5 than Models 3 and 4, independent of osteoporosis. Even in the instance of endplate damage, the Model 2 was subjected to less force than the Models 3 and 4 that used the short cage design.

## Discussion

In recent years, treating degenerative lumbar disorders with minimally invasive surgery has grown in popularity. The common surgical procedure used nowadays is called Lateral Lumbar Interbody Fusion (LLIF). Two surgical approaches can be used: 1) extreme lateral interbody fusion through the psoas major muscle into the intervertebral disc (Ozgur et al., 2006); 2) Lateral interbody fusion via the oblique corridor between the aorta and the psoas major muscle (Silvestre et al., 2012). The surgical procedure of interbody fusion still poses difficulties for surgeons due to the incidence of postoperative problems, despite major advances in medical technology. Non-fusion, pseudarthrosis development, and prosthesis sinking are some of these adverse effects. We performed a biomechanical analysis using finite element analysis to compare the utilization of a long cage with a short cage in the event of endplate failure. We also looked at patient models for osteoporosis. Our research suggests that using a lengthy cage over the lumbar cricoid process can have a number of benefits. These include higher fusion rates and a lower chance of issues such cage subsidence, a screw-rod internal fixation method that fails, and neighboring segment degeneration. Even when patients have osteoporosis and the surgical technique results in iatrogenic endplate destruction, these advantages still apply.

The ROM of the fused segment in the postoperative model displayed varying behavior depending on cage architecture, endplate destruction, and bone material qualities (Normal/Osteoporotic) under a combined load of 280N vertical load and 7.5 nm torque. First off, Model 4Osteoporotic has the greatest ROM (1.15°) out of the eight models. ROM less than 5° was regarded as effective interbody fusion in accordance with the FDA’s definition of the term. As a result, we assumed that all models had a stable fusion following surgery. However, as seen in Figure 6, there were some noticeable differences amongst the eight models. The long cage demonstrated better restraint capacity in both the normal and osteoporotic animals, and this capacity remained mostly constant even in the presence of endplate loss. On the other hand, when the endplate was healthy, the ROM of the short cage model grew by 9.4%–44.97%, and by 14%–51.68% when the endplate was damaged. This suggests that endplate damage may cause an early rise in the fused segment’s range of motion (ROM) after surgery, which may result in long-term postoperative problems. In our study, individuals with osteoporosis, particularly those who had endplate damage, showed a larger improvement in range of motion. In comparison to individuals without osteoporosis, the ROM of the fused segment exhibited a considerable increase in all directions, with the largest increase reaching 288.13%. Our findings are consistent with those of Bereczki et al. (2021), where they also examined osteoporotic and normal models using OLIF (Oblique Lateral Interbody Fusion) and various immobilization techniques. In contrast to people without osteoporosis, their study showed that patients with the condition had a greater range of motion in all directions. Our research shows that endplate damage in osteoporotic individuals greatly increases the instability of fusion segment fixation. However, this instability can be successfully reduced by using a cage that spans the lumbar annular process.

The loss of normal mobility brought on by the stiff immobilization of the motion segment might trigger compensatory increases in the motion and intradiscal pressure of neighbouring segments. In turn, this quickens degeneration and raises the possibility of neighboring segment illness (Song et al., 2011; Hekimoglu et al., 2021). When compared to the normal models in our investigation, the postoperative models with normal bone quality showed comparable or even less mobility in the neighboring segments. Even less mobility than in the normal models was present in the sagittal and axial planes. In comparison to models with normal bone quality and intact preoperative models, the mobility of neighboring segments was greater in the osteoporotic models but the difference overall was not statistically significant. In addition, none of the eight postoperative models’ intra-discal pressures, as seen in Figure 8, significantly differed from one another. Taking into account earlier research on the risk factors for adjacent segment illness, it has been discovered that the key predictors of adjacent segment disease are things like decompression of the non-fused segment, the level of the fused segment, and the degree of degeneration in the neighboring segments. The choice of surgical technique, the use of pedicle screws and fusion devices, and the incidence of neighboring segment disease are not considerably increased (Natarajan and Andersson, 2017; Maragkos et al., 2020). Therefore, in our study, the use of both types of cages, regardless of endplate integrity or the presence of osteoporosis, had minimal impact on adjacent segment degeneration.

An interbody fusion cage and screw-rod system provide a reliable stress transmission channel within the internal fixation system, according to prior research (Han et al., 2021). Applying an interbody fusion cage to the anterior column efficiently distributes pressure there and lessens stress on the screw-rod system. This idea was further reinforced by Wu et al.'s investigation, which revealed a decreasing trend in screw-rod system stress levels as the cage’s axial area rose in the postoperative model. The bigger contact area between the Long Cage and the endplate in our investigation greatly increased the anterior column’s ability to support loads. The Long Cage also uses the robustness of the lumbar annular process on both sides to span across it, thus releasing pressure inside the internal fixation system. In comparison to other models, the stress experienced by the screen-rod system was significantly decreased, even in the presence of endplate damage and osteoporosis. Although the highest stress measured in the screw-rod system of the postoperative model was only 117.6 MPa, much less than the titanium metal’s yield strength (825–895 MPa) (Liang et al., 2020), it is important to take into account the compromised bone quality in osteoporotic individuals. The screen-vertebral contact also acts as a conduit for the tension inside the screw-rod system. Greater pressure on the vertebral body is implied by greater tension on the screw-rod system. In order to reduce the likelihood of internal fixation failure and screw rod fractures, it is advised to reduce total load on the internal fixation system given the complex and dynamic nature of everyday activities.

**FIGURE 11:**
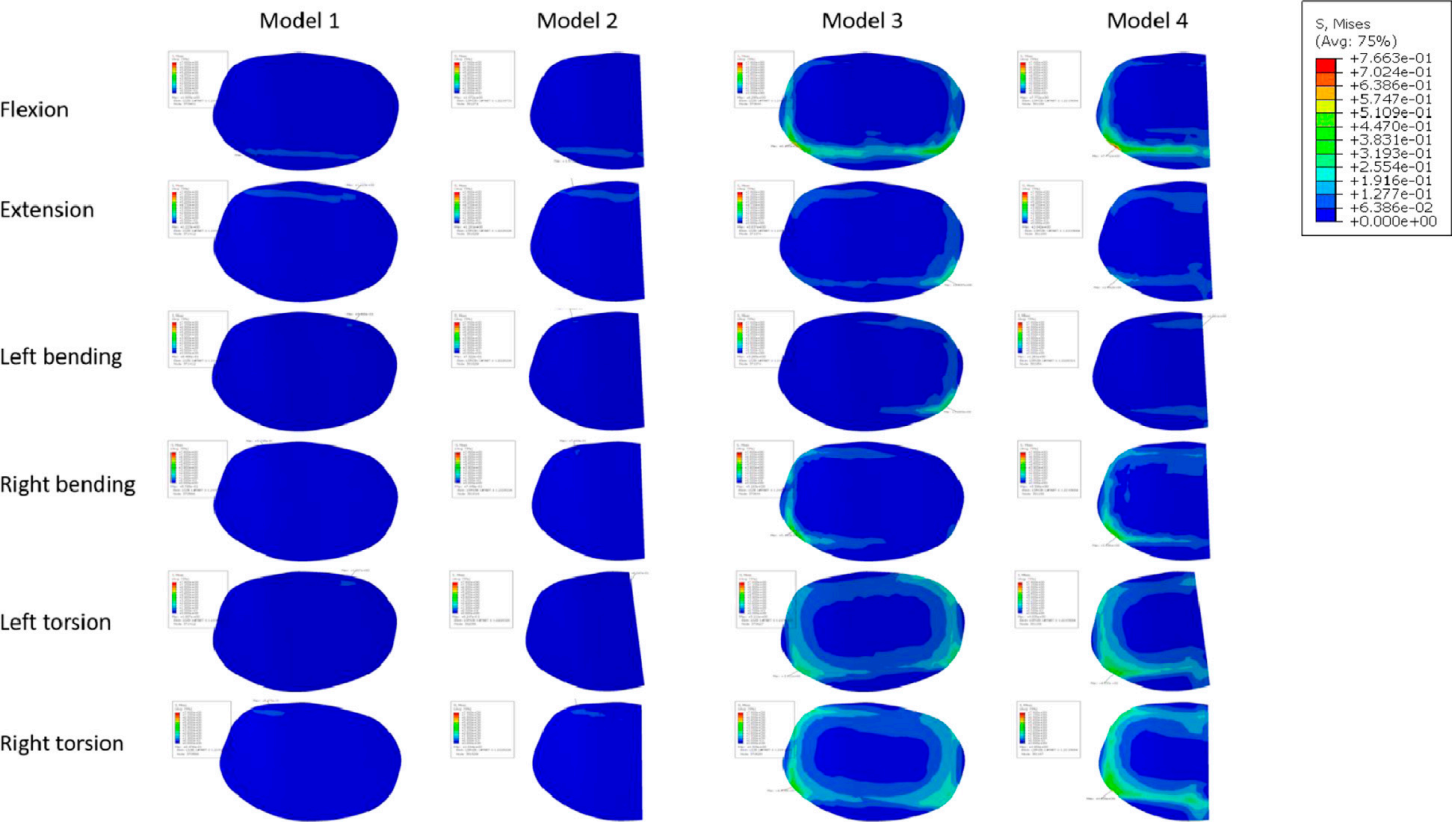
Endplate stress map of normal bone quality model.

**FIGURE 12:**
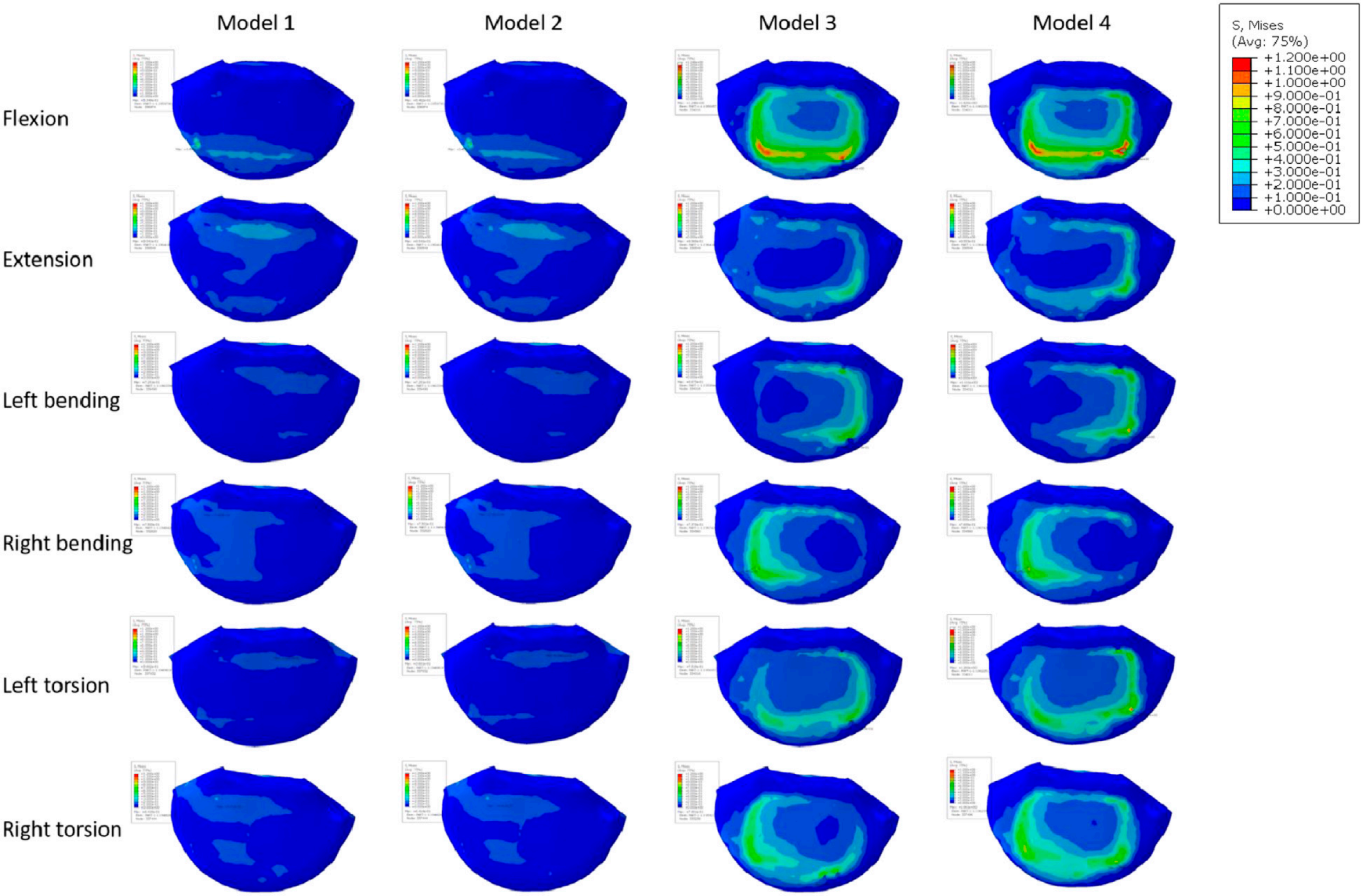
Stress map of cancellous bone on the upper surface of L5 in the osteoporotic model.

Numerous studies have universally acknowledged the importance of the endplate. The structural qualities of the lumbar vertebral body can be significantly reduced by the removal of the endplate (Hou et al., 2013; Oh et al., 2017). In spinal fusion, a number of variables, including LLIF, have been found to cause cage sinking. Risk elements for cage sinking may include advanced age, female gender, larger cage, multi-level instances, and osteoporosis (Alkalay et al., 2018). Most biomechanical stability evaluation studies preserve the endplate’s integrity throughout preparation and testing while excluding specimens with severe osteoporosis. However, intraoperative endplate violation during intervertebral disc ectomy may occur, impacting segmental stiffness, subsidence, and maybe fusion rates in surgical patients with inferior bone quality (Briski et al., 2017). The force on the cage’s upper surface becomes a crucial consideration in preventing cage collapse when endplate damage occurs. In order to do so, we shall talk about the stress on the endplate and cancellous bone, and Figures 11, 12 depicts the stress map. In order to test the biomechanical performance of cages spanning the vertebral ring apophysis in the context of simulated endplate injury, we simulated and analyzed models of individuals with normal bone quality and osteoporosis. In our findings, the maximum trabecular bone stress rose by 0.12 MPa and the maximum endplate stress increased by 0.17 MPa in Model 2 of the normal bone quality model in comparison to Model 1. Although there was no statistically significant difference in the maximum trabecular bone stress between the two models, the average maximum trabecular bone stress in Models 1 and 2 of the osteoporotic model (0.28 MPa) was slightly higher than that in the normal bone quality model (0.23 MPa) in the osteoporotic model. Model 2 also showed a maximum increase in endplate stress compared to Model 1 of 0.46 MPa. Additionally, as compared to Model 3, Model 4 exhibited a maximum increase in endplate stress of 1.9 MPa and a maximum increase in trabecular bone stress of 0.92 MPa in the normal bone quality model. When compared to Model 3, Model 4 showed a maximum increase in endplate stress of 2.5 MPa and a maximum increase in trabecular bone stress of 0.6 MPa in the osteoporotic model. In conclusion, the use of a Long cage model can lessen stress on the top surface of the L5 endplate and nearby trabecular bone, independent of the existence of endplate deterioration or osteoporosis. Our findings are in agreement with those of Briski et al. (Briski et al., 2017) in their cadaveric study, Thus they came to the conclusion that, independent of the integrity of the endplate or the presence of osteoporosis, bigger cages across the endplate ring apophysis can increase compressive strength and lower cage sinking at the operational level.

Our study has a number of drawbacks. First off, there was no statistical analysis done on the data in this study because it was taken from a single spinal model of a 28-year-old male adult, which raises the likelihood of individual variances. This is a typical finite element analysis limitation. Additionally, we simplified the cage model by assuming full fusion between the cage and the surrounding vertebral bodies, as well as the material characteristics of the model by assuming isotropy for each component. In the future, we want to concentrate on the unique material characteristics and the surface shape of the cage. Furthermore, we did not mimic the difficulties associated with muscle alterations, which would more accurately reflect the physiological features of the typical lumbar spine. Second, although the osteoporosis population was taken into account, our study was only able to use one osteoporotic model. It is challenging to properly incorporate these aspects in a single model since the severity of osteoporosis can change greatly across different individuals and structural variations like osteophytes or disc degeneration tend to speed up when osteoporosis is present. This is a restriction that we are aware of. Thirdly, the stress conditions on the facet joints were not taken into account in our investigation. Future investigations with independent models are planned to address this. Fourthly, in this study, the use of a 30 mm “short cage” is indeed uncommon in clinical practice. However, this cage size was chosen to precisely cover the area above the endplate to ensure the accuracy of the results. In subsequent research, we plan to increase the endplate area to better align with real-world scenarios. Furthermore, the perfect fusion of the cage with the vertebra, as achieved in this study through Boolean operations, is also relatively uncommon in clinical settings. In future research, we intend to introduce conditions that include imperfect cage-vertebra contact to better reflect the clinical reality. And due to our center’s exclusive utilization of the aforementioned internal fixation method, the potential influence of various internal fixation approaches on the outcomes has not been explored. Particularly in the case of osteoporotic patients, a more optimal nail placement technique could potentially reduce stress on the fusion device, thereby promoting fusion more effectively. We intend to carry out more thorough and rigorous biomechanical research in the future to confirm our findings.

## Conclusion

The endplate needs to be safeguarded during the procedure since it is a crucial element that will support the cage after the operation. The use of a trans-intervertebral fusion cage that is 5 mm longer than the length of the bilateral pedicle as determined by preoperative X-ray might lessen stress on the internal fixation system and endplate, regardless of whether the endplate is intact or osteoporosis is present. The disc pressure or range of motion in adjacent segments are not increased by this strategy. When there is endplate degeneration or osteoporosis, using a smaller cage may put additional strain on the entire fixation system and raise the chance of complications.

## Data Availability

The raw data supporting the conclusions of this article will be made available by the authors, without undue reservation.

## References

[B1] Alimi M. Lang G. Navarro-Ramirez R. Perrech M. Berlin C. Hofstetter C. P. (2018). The impact of cage dimensions, positioning, and side of approach in extreme lateral interbody fusion. Clin. Spine Surg. 31 (1), E42–E49. 10.1097/bsd.0000000000000507 28319468

[B2] Alizadeh M. Kadir M. R. Fadhli M. M. Fallahiarezoodar A. Azmi B. Murali M. R. (2013). The use of X-shaped cross-link in posterior spinal constructs improves stability in thoracolumbar burst fracture: a finite element analysis. J. Orthop. Res. 31 (9), 1447–1454. 10.1002/jor.22376 23640802

[B3] Alkalay R. N. Adamson R. Groff M. W. (2018). The effect of interbody fusion cage design on the stability of the instrumented spine in response to cyclic loading: an experimental study. Spine J. 18 (10), 1867–1876. 10.1016/j.spinee.2018.03.003 29526639

[B4] Bereczki F. Turbucz M. Kiss R. Eltes P. E. Lazary A. (2021). Stability evaluation of different oblique lumbar interbody fusion constructs in normal and osteoporotic condition - a finite element based study. Front. Bioeng. Biotechnol. 9, 749914. 10.3389/fbioe.2021.749914 34805108 PMC8602101

[B5] Brinckmann P. Grootenboer H. (1991). Change of disc height, radial disc bulge, and intradiscal pressure from discectomy. An *in vitro* investigation on human lumbar discs. Spine (Phila Pa 1976) 16 (6), 641–646. 10.1097/00007632-199106000-00008 1862403

[B6] Briski D. C. Goel V. K. Waddell B. S. Serhan H. Kodigudla M. K. Palepu V. (2017). Does spanning a lateral lumbar interbody cage across the vertebral ring apophysis increase loads required for failure and mitigate endplate violation. Spine (Phila Pa 1976) 42 (20), E1158–E1164. 10.1097/brs.0000000000002158 28472018

[B7] Buser Z. Tekmyster G. Licari H. Lantz J. M. Wang J. C. (2021). Team approach: management of an acute L4-L5 disc herniation. JBJS Rev. 9 (10). 10.2106/jbjs.rvw.21.00003 34637405

[B8] Cho A. R. Cho S. B. Lee J. H. Kim K. Y. (2015). Effect of augmentation material stiffness on adjacent vertebrae after osteoporotic vertebroplasty using finite element analysis with different loading methods. Pain Physician 18 (6), E1101–E1110. 10.36076/ppj.2015/18/e1101 26606023

[B9] Choi J. Shin D. A. Kim S. (2017). Biomechanical effects of the geometry of ball-and-socket artificial disc on lumbar spine: a finite element study. Spine (Phila Pa 1976) 42 (6), E332–E339. 10.1097/brs.0000000000001789 27428389

[B10] Elowitz E. H. (2015). Central and foraminal indirect decompression in MIS lateral interbody fusion (XLIF): video lecture. Eur. Spine J. 24 (3), 449–450. 10.1007/s00586-015-3946-6 25904414

[B11] Grant J. P. Oxland T. R. Dvorak M. F. (2001). Mapping the structural properties of the lumbosacral vertebral endplates. Spine (Phila Pa 1976) 26 (8), 889–896. 10.1097/00007632-200104150-00012 11317111

[B12] Grant J. P. Oxland T. R. Dvorak M. F. Fisher C. G. (2002). The effects of bone density and disc degeneration on the structural property distributions in the lower lumbar vertebral endplates. J. Orthop. Res. 20 (5), 1115–1120. 10.1016/s0736-0266(02)00039-6 12382980

[B13] Han X. Chen X. Li K. Li Z. Li S. (2021). Finite analysis of stability between modified articular fusion technique, posterior lumbar interbody fusion and posteriorlateral lumbar fusion. BMC Musculoskelet. Disord. 22 (1), 1015. 10.1186/s12891-021-04899-x 34863121 PMC8645152

[B14] Hekimoglu M. Basak A. Yilmaz A. Yıldırım H. Aydın A. L. Karadag K. (2021). Adjacent segment disease (ASD) in incidental segmental fused vertebra and comparison with the effect of stabilization systems on ASD. Cureus 13 (10), e18647. 10.7759/cureus.18647 34786242 PMC8578681

[B15] Hou Y. Yuan W. Kang J. Liu Y. (2013). Influences of endplate removal and bone mineral density on the biomechanical properties of lumbar spine. PLoS One 8 (11), e76843. 10.1371/journal.pone.0076843 24244269 PMC3820638

[B16] Huang Y. P. Du C. F. Cheng C. K. Zhong Z. C. Chen X. W. Wu G. (2016). Preserving posterior complex can prevent adjacent segment disease following posterior lumbar interbody fusion surgeries: a finite element analysis. PLoS One 11 (11), e0166452. 10.1371/journal.pone.0166452 27870867 PMC5117648

[B17] Kang S. Park C. H. Jung H. Lee S. Min Y. S. Kim C. H. (2022). Analysis of the physiological load on lumbar vertebrae in patients with osteoporosis: a finite-element study. Sci. Rep. 12 (1), 11001. 10.1038/s41598-022-15241-3 35768481 PMC9243026

[B18] Kim H. J. Kang K. T. Chang B. S. Lee C. K. Kim J. W. Yeom J. S. (2014). Biomechanical analysis of fusion segment rigidity upon stress at both the fusion and adjacent segments: a comparison between unilateral and bilateral pedicle screw fixation. Yonsei Med. J. 55 (5), 1386–1394. 10.3349/ymj.2014.55.5.1386 25048501 PMC4108828

[B19] Kim Y. H. Ha K. Y. Kim K. T. Chang D. G. Park H. Y. Yoon E. J. (2021). Risk factors for intraoperative endplate injury during minimally-invasive lateral lumbar interbody fusion. Sci. Rep. 11 (1), 20149. 10.1038/s41598-021-99751-6 34635757 PMC8505407

[B20] Kotheeranurak V. Jitpakdee K. Lin G. X. Mahatthanatrakul A. Singhatanadgige W. Limthongkul W. (2021). Subsidence of interbody cage following oblique lateral interbody fusion: an analysis and potential risk factors. Glob. Spine J. 13, 1981–1991. 10.1177/21925682211067210 PMC10556923 34920690

[B21] Liang Z. Cui J. Zhang J. He J. Tang J. Ren H. (2020). Biomechanical evaluation of strategies for adjacent segment disease after lateral lumbar interbody fusion: is the extension of pedicle screws necessary? BMC Musculoskelet. Disord. 21 (1), 117. 10.1186/s12891-020-3103-1 32085708 PMC7035718

[B22] Li C. Zhou Y. Wang H. Liu J. Xiang L. (2014). Treatment of unstable thoracolumbar fractures through short segment pedicle screw fixation techniques using pedicle fixation at the level of the fracture: a finite element analysis. PLoS One 9 (6), e99156. 10.1371/journal.pone.0099156 24914815 PMC4051693

[B23] Liu C. W. Wang L. L. Xu Y. K. Chen C. M. Wang J. C. Tsai W. T. (2020). Traditional and cortical trajectory screws of static and dynamic lumbar fixation-a finite element study. BMC Musculoskelet. Disord. 21 (1), 463. 10.1186/s12891-020-03437-5 32664920 PMC7362474

[B24] Lu T. Lu Y. (2019). Comparison of biomechanical performance among posterolateral fusion and transforaminal, extreme, and oblique lumbar interbody fusion: a finite element analysis. World Neurosurg. 129, e890–e899. 10.1016/j.wneu.2019.06.074 31226452

[B25] Malham G. M. Parker R. M. Blecher C. M. Seex K. A. (2015). Assessment and classification of subsidence after lateral interbody fusion using serial computed tomography. J. Neurosurg. Spine 23 (5), 589–597. 10.3171/2015.1.spine14566 26207320

[B26] Maragkos G. A. Motiei-Langroudi R. Filippidis A. S. Glazer P. A. Papavassiliou E. (2020). Factors predictive of adjacent segment disease after lumbar spinal fusion. World Neurosurg. 133, e690–e694. 10.1016/j.wneu.2019.09.112 31568911

[B27] Marchi L. Abdala N. Oliveira L. Amaral R. Coutinho E. Pimenta L. (2013). Radiographic and clinical evaluation of cage subsidence after stand-alone lateral interbody fusion. J. Neurosurg. Spine 19 (1), 110–118. 10.3171/2013.4.spine12319 23662890

[B28] Natarajan R. N. Andersson G. B. (2017). Lumbar disc degeneration is an equally important risk factor as lumbar fusion for causing adjacent segment disc disease. J. Orthop. Res. 35 (1), 123–130. 10.1002/jor.23283 27152925

[B29] Obenchain T. G. (1991). Laparoscopic lumbar discectomy: case report. J. Laparoendosc. Surg. 1 (3), 145–149. 10.1089/lps.1991.1.145 1836399

[B30] Oh K. W. Lee J. H. Lee J. H. Lee D. Y. Shim H. J. (2017). The correlation between cage subsidence, bone mineral density, and clinical results in posterior lumbar interbody fusion. Clin. Spine Surg. 30 (6), E683–E689. 10.1097/bsd.0000000000000315 28632554

[B31] Oxland T. R. Grant J. P. Dvorak M. F. Fisher C. G. (2003). Effects of endplate removal on the structural properties of the lower lumbar vertebral bodies. Spine (Phila Pa 1976) 28 (8), 771–777. 10.1097/01.brs.0000060259.94427.11 12698119

[B32] Ozgur B. M. Aryan H. E. Pimenta L. Taylor W. R. (2006). Extreme Lateral Interbody Fusion (XLIF): a novel surgical technique for anterior lumbar interbody fusion. Spine J. 6 (4), 435–443. 10.1016/j.spinee.2005.08.012 16825052

[B33] Park W. M. Kim K. Kim Y. H. (2013). Effects of degenerated intervertebral discs on intersegmental rotations, intradiscal pressures, and facet joint forces of the whole lumbar spine. Comput. Biol. Med. 43 (9), 1234–1240. 10.1016/j.compbiomed.2013.06.011 23930818

[B34] Polikeit A. Ferguson S. J. Nolte L. P. Orr T. E. (2003). Factors influencing stresses in the lumbar spine after the insertion of intervertebral cages: finite element analysis. Eur. Spine J. 12 (4), 413–420. 10.1007/s00586-002-0505-8 12955610 PMC3467788

[B35] Polly D. W. JR. Klemme W. R. Cunningham B. W. Burnette J. B. Haggerty C. J. Oda I. (2000). The biomechanical significance of anterior column support in a simulated single-level spinal fusion. J. Spinal Disord. 13 (1), 58–62. 10.1097/00002517-200002000-00012 10710152

[B36] Renner S. M. Natarajan R. N. Patwardhan A. G. Havey R. M. Voronov L. I. Guo B. Y. (2007). Novel model to analyze the effect of a large compressive follower pre-load on range of motions in a lumbar spine. J. Biomech. 40 (6), 1326–1332. 10.1016/j.jbiomech.2006.05.019 16843473

[B37] Schmidt H. Heuer F. Drumm J. Klezl Z. Claes L. Wilke H. J. (2007). Application of a calibration method provides more realistic results for a finite element model of a lumbar spinal segment. Clin. Biomech. (Bristol, Avon) 22 (4), 377–384. 10.1016/j.clinbiomech.2006.11.008 17204355

[B38] Sengul E. Ozmen R. Yaman M. E. Demir T. (2021). Influence of posterior pedicle screw fixation at L4-L5 level on biomechanics of the lumbar spine with and without fusion: a finite element method. Biomed. Eng. Online 20 (1), 98. 10.1186/s12938-021-00940-1 34620170 PMC8499536

[B39] Silvestre C. Mac-Thiong J. M. Hilmi R. Roussouly P. (2012). Complications and morbidities of mini-open anterior retroperitoneal lumbar interbody fusion: oblique lumbar interbody fusion in 179 patients. Asian Spine J. 6 (2), 89–97. 10.4184/asj.2012.6.2.89 22708012 PMC3372554

[B40] Song K. J. Choi B. W. Jeon T. S. Lee K. B. Chang H. (2011). Adjacent segment degenerative disease: is it due to disease progression or a fusion-associated phenomenon? Comparison between segments adjacent to the fused and non-fused segments. Eur. Spine J. 20 (11), 1940–1945. 10.1007/s00586-011-1864-9 21656051 PMC3207329

[B41] Song S. Guo Y. Yang Y. Fu D. (2022). Advances in pathogenesis and therapeutic strategies for osteoporosis. Pharmacol. Ther. 237, 108168. 10.1016/j.pharmthera.2022.108168 35283172

[B42] Su Q. Li C. Li Y. Zhou Z. Zhang S. Guo S. (2020). Analysis and improvement of the three-column spinal theory. BMC Musculoskelet. Disord. 21 (1), 537. 10.1186/s12891-020-03550-5 32787828 PMC7425572

[B43] Su Y. Wang X. Ren D. Liu Y. Liu S. Wang P. (2018). A finite element study on posterior short segment fixation combined with unilateral fixation using pedicle screws for stable thoracolumbar fracture. Med. Baltim. 97 (34), e12046. 10.1097/md.0000000000012046 PMC6112892 30142856

[B44] Takenaka S. Kaito T. Ishii K. Watanabe K. Watanabe K. Shinohara A. (2020). Influence of novel design alteration of pedicle screw on pull-out strength: a finite element study. J. Orthop. Sci. 25 (1), 66–72. 10.1016/j.jos.2019.03.002 30902538

[B45] Walker C. T. Farber S. H. Cole T. S. Xu D. S. Godzik J. Whiting A. C. (2019). Complications for minimally invasive lateral interbody arthrodesis: a systematic review and meta-analysis comparing prepsoas and transpsoas approaches. J. Neurosurg. Spine 30, 446–460. 10.3171/2018.9.spine18800 30684932

[B46] Wang Z. Ma R. Cai Z. Yang S. Ge Z. (2021). Biomechanical evaluation of stand-alone oblique lateral lumbar interbody fusion under 3 different bone mineral density conditions: a finite element analysis. World Neurosurg. 155, e285–e293. 10.1016/j.wneu.2021.08.049 34418606

[B47] Weinstein J. N. Spratt K. F. Spengler D. Brick C. Reid S. (1988). Spinal pedicle fixation: reliability and validity of roentgenogram-based assessment and surgical factors on successful screw placement. Spine (Phila Pa 1976) 13 (9), 1012–1018. 10.1097/00007632-198809000-00008 3206294

[B48] Wu J. Yang D. Han Y. Xu H. Wen W. Xu H. (2022). Application of dual-trajectory screws in revision surgery for lumbar adjacent segment disease: a finite element study. J. Orthop. Surg. Res. 17 (1), 427. 10.1186/s13018-022-03317-9 36153558 PMC9509616

[B49] Yuan W. Kaliya-Perumal A. K. Chou S. M. Oh J. Y. L. (2020). Does lumbar interbody cage size influence subsidence? A biomechanical study. Spine (Phila Pa 1976) 45 (2), 88–95. 10.1097/brs.0000000000003194 31415458

[B50] Zeng Z. Y. Xu Z. W. He D. W. Zhao X. Ma W. Ni W. (2018). Complications and prevention strategies of oblique lateral interbody fusion technique. Orthop. Surg. 10 (2), 98–106. 10.1111/os.12380 29878716 PMC6594526

[B51] Zhao X. Du L. Xie Y. Zhao J. (2018). Effect of lumbar lordosis on the adjacent segment in transforaminal lumbar interbody fusion: a finite element analysis. World Neurosurg. 114, e114–e120. 10.1016/j.wneu.2018.02.073 29477002

